# Peptidomimetics Based On Dehydroepiandrosterone Scaffold: Synthesis, Antiproliferation Activity, Structure-Activity Relationship, and Mechanisms

**DOI:** 10.1038/srep32654

**Published:** 2016-09-02

**Authors:** Xiaohui Wang, Haihuan Su, Wenda Wang, Changshui Chen, Xiufang Cao

**Affiliations:** 1 College of Science, Huazhong Agricultural University, Wuhan, 430070, China

## Abstract

A series of novel peptidomimetics bearing dehydroepiandrosterone moiety were designed, synthesized, and evaluated for their inhibition activities against cell proliferation. According to the preliminary studies on inhibitory activities, some of the newly prepared compounds indicated significantly inhibition activities against human hepatoma cancer (HepG2), human lung cancer (A549), human melanoma (A875) cell lines compared with the control 5-fluorouracil. Especially, compounds **Ii** (IC_50_ < 14 μM) and **Ik** (IC_50_ < 13 μM) exhibited obvious inhibition activities against all tested cell lines. The highly potential compound **Ik** induced apoptosis in HepG2 cells were analyzed by flow cytometry, and the apoptotic effects of compound **Ik** were further evaluated using Annexin V-FITC/propidium iodide dual staining assay, which revealed these highly potential compounds induced cell death in HepG2 cells at least partly by apoptosis.

Cancers is the leading cause of morbidity and mortality worldwide, with approximately 14 million new cases and 8.2 million cancer related deaths in 2012^1^. The number of new cases is expected to rise by about 70% over the next 2 decades^2^. Searching and developing effective anticancer drugs is more and more important. Peptides and their derivatives are important molecules with versatile pharmacological properties^3^, and which are normally designed to mimic a natural protein or peptide. Nevertheless, stability and bioavailability of peptides and mimetics can be improved by several modifications^4^. In addition, some dipeptide derivatives have exhibited potent inhibition activities against human tumors cells^5^.

Besides that, steroids are a class of typical lipids found in living world that have broad biological activities^6^, and which have been widely used in medicine as essentials of anti-inflammatory, anabolic, anticancer and contraceptive drugs^7^. Recent years, the modifications of natural steroid have become a focus of research probably on account of the various advantages associated with steroid based chemotherapeutics. Dehydroepiandrosterone (DHEA) is a major steroid secreted by the adrenal gland and the most abundant steroid in humans^8^. Furthermore, several steroidal derivatives have been investigated as potential anti-cancer agents for the treatment of breast cancer, prostate cancer, ovary cancer, lung cancer, gastric cancer, esophageal cancer, heptoma cancer, melanoma cancer, multiple myeloma^9^,^10^,^11^,^12^,^13^,^14^,^15^,^16^,^17^,^18^. On the other hand, structural modifications carried out at positions 17 of DHEA have exhibited a broad range biological activities as potent antimicrobial agents and anticancer agents^10^,^19^.

Recently, during the course of our research for high active compounds, three series of novel peptidomimetics bearing natural tryptamine moiety were designed, synthesized, and evaluated for their inhibition activities against cell proliferation^20^. Some of the prepared compounds exhibited significant inhibition activities against human hepatoma cancer (HepG2 and Huh-7), human melanoma (A875) cell lines compared with the control 5-fluorouracil. The results from these investigations inspired us to further investigate the novel amino acid-conjugates of dehydroepiandrosterone, which adopt the natural DHEA scaffold to replace the natural tryptamine moiety (Fig. 1). To study the possible structure-activity relationships, several efforts in structure modifications of such type of compounds were designed, and the synthesis of target compounds is simple and convenient as shown in Fig. 2. Besides, their inhibition activities against various cancer cell lines (HepG2, A549, and A875) were also evaluated by MTT method, and the possible mechanism of action for the highly potential compounds were also evaluated using Annexin V-FITC/propidium iodide dual staining assay.

## Results and Discussion

### Chemistry

**Figure 2:**
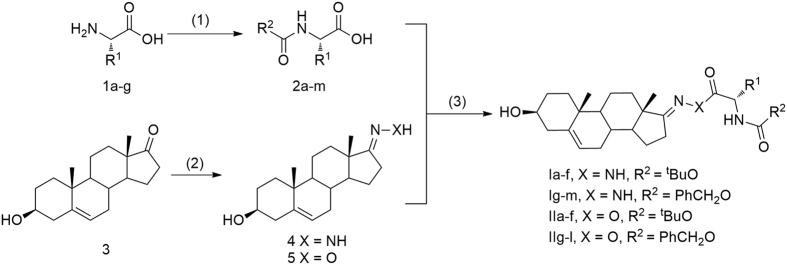
Synthetic route and conditions for target compounds. (1) (Boc)_2_O or CbzCl, NaOH, 0 ^o^C to rt, yields 65–80%; (2) NH_2_NH_2_·H_2_O, EtOH (X = NH) or NH_2_OH·HCl, CH_3_COONa, EtOH (X = O), reflux, yields 80–85%; (3) CDI, Et_3_N, MeCN, rt, yields 50–80%.

In the present study, a series of peptidomimetics including steroids groups were designed and synthesized in a simple and convenient route. The general synthetic method for all compounds is outlined in Fig. 2.

The easily available amino acids **1a-g** was selected as staring materials, and which were transferred to the corresponding *N*-(tert-butoxycarbonyl)-amino acids and *N*-benzyloxycarbonyl-amino acids **2a-m** by electrophilic substitution reactions. Meanwhile, compounds dehydroepiandrosterone-17 hydrazone **4** and dehydroepiandrosterone-17 oxime **5** were conveniently prepared from dehydroepiandrosterone **3** by nucleophilic addition elimination reactions. Then the desired peptidomimetics **Ia-m** were obtained from N-protected amino acids **2a-m** and dehydroepiandrosterone-17 hydrazone **4** by nucleophilic substitution. Similarly, compounds **IIa-l** were also obtained from N-protected amino acids **2a-m** with dehydroepiandrosterone-17 oxime **5** by nucleophilic substitution as well. All the compounds gave satisfactory chemical analyses, and the chemical structures and physiochemical properties of the synthesized compounds were summarized in Table 1.

**Figure 3:**
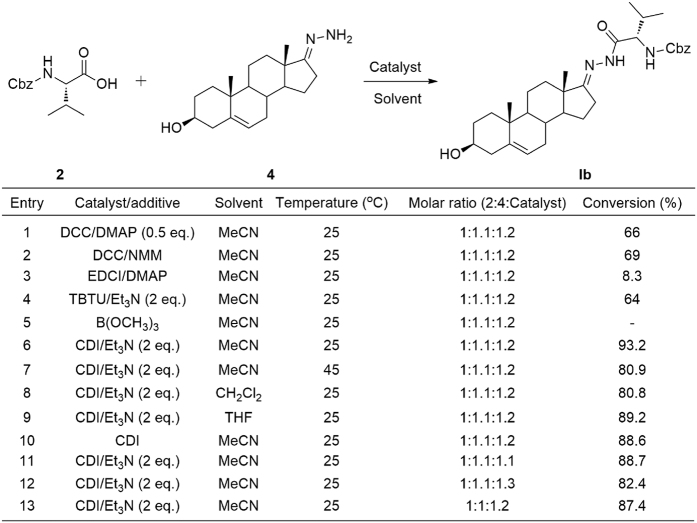
Reaction conditions screening.

Although the condensation reactions between carboxylic acid and RNH_2_/ROH can be generated by a lot of catalysts, we wish to develop convenient and effective methods for our own syntheses. First, compound dehydroepiandrosterone-17 hydrazone **4** and N-cbz-L-valine was chosen as a model system (Fig. 3). Six kinds of common and appropriate catalyst composition were examined and screened, dicyclohexylcarbodiimide and 4-dimethylaminopyridine (DCC/DMAP), dicyclohexylcarbodiimide and 4-methylmorpholine (DCC/NMM), 1-ethyl-3-(3-dimethylaminopropyl)carbodiimide hydrochloride and 4-dimethylaminopyridine (EDCI/DMAP), O-(benzotriazol-1-yl)-N,N,N’,N’-tetramethyluronium tetrafluoroborate and triethylamine (TBTU/Et_3_N), trimethyl borate (B(OCH_3_)_3_), N,N’-carbonyldiimidazole and triethylamine (CDI/Et_3_N) (Fig. 3, Entries **1–6**). CDI and Et_3_N composition was found to give the best conversion. Besides, we found high temperature cannot improve the conversion (Fig. 3, Entries **6, 7**). Different solvents were screened in order to increase the conversion (Fig. 3, Entries **6, 8, 9**). Acetonitrile was found to give the best conversion among the three solvents. Without triethylamine, the conversion has a little decrease (Fig. 3, Entry **10**). Furthermore, different molar ratios for the substrates were also examined to increase the conversion (Fig. 3, Entries **6, 11, 12**, and **13**). The best condition was shown in Fig. 3 as entry 6 in summary.

### Inhibitory effects of compounds on the proliferation of various cancer cells

**Figure 4:**
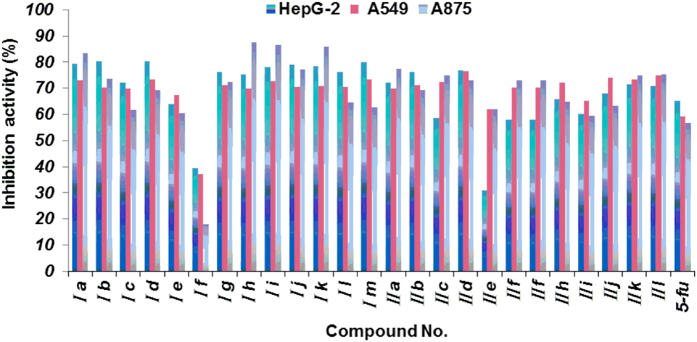
Inhibition activities against cell proliferation for target compounds at 40 μg/mL. Abbreviations: HepG2 - Human hepatocellular liver carcinoma cell line; A549 -Human lung cell line; A875 - Human melanoma cell line; 5-FU - 5-Fluorouracil, used as a positive control.

The newly prepared peptidomimetics derivatives were evaluated for their *in vitro* cytotoxic effects against HepG2 (hepatocellular liver carcinoma), A549 (Human lung cell line), A875 (human melanoma cell line) by the standard MTT (3-(4,5-dimethylthiazol-2-yl)-2,5-diphenyl tetrazolium bromide) assay^21^ using 5-FU (5-Fluorouracil) as a positive control. The preliminary results were summarized in Fig. 4 and Table 2. The IC_50_ value represents the drug concentration required to inhibit cell growth by 50%.

Generally, as shown in Fig. 4, the prepared peptidomimetics derivatives (1–26) showed moderate to good inhibition activities against the three tested human cancer cell lines. Most compounds displayed better inhibition activities than 5-FU. Notably, the compounds **Ia**, **Ib**, **Ii**, **Ij**, **Ik, Il**, and **IId**, **IIk**, **IIl** exhibited significant inhibitory activities against all three tested cell lines with 70.1–86.4% growth inhibition at 40 μg/mL concentration compared to the positive control 5-FU (56.6–65.3%). Also, it is interesting to note that compound **IIe** showed selective cytotoxicity to A549 cell line and A875 cell line with 62.1% and 62% growth inhibition respectively, and with 31% inhibitions to HepG2 cell lines.

Moreover, the preliminary bioassay indicated that most of the target compounds (such as **Ia**, **Ib**, **Ii**, **Ij**, **Ik**, and **IId**, **IIk** and **IIl**) displayed good inhibitory activities compared to 5-FU, so in order to investigate the potential activities, the IC_50_ values were further evaluated. The inhibitory activities expressed as IC_50_ values for the target compounds are presented in Table 2. The results also testify that some of the designed peptidomimetics derivatives exhibited higher inhibition activity than the control 5-FU under the same conditions. As indicated in Table 2, compound **IId** showed the strongest inhibitory effect against HepG2, with an IC_50_ value of 7 μM; compound **Ik** showed the strongest inhibitory effect against A549 and A875 with an IC_50_ value of 6 and 13 μM, respectively. We also can find that compound **Ib** have the same inhibition activities trend as 5-FU against the three cancer cell lines. Especially, compounds **Ib, Ic, Ii**, **Ik, Il, Im, IId** and **IIk** exhibited significant inhibition against all tested cancer cell lines compared to the positive control 5-FU.

**Figure 5:**
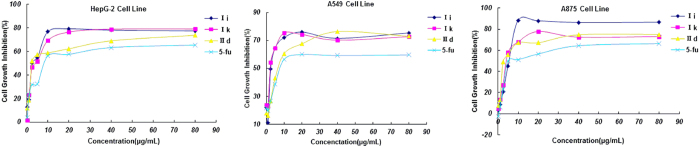
Dose–response analysis of cell growth inhibitory activity for representative compounds **Ii**, **Ik**, **IId** and 5-FU (positive control) against HepG2 (left), A549 (middle) and A875(right) cell lines.

Furthermore, the dose-response analysis of cell growth inhibition activity for representative compounds **Ii**, **Ik**, **IId** and 5-FU has been displayed in Fig. 5, which revealed that the cytotoxic effects on cell lines of target compounds indicated obvious concentration-dependent manner.

### Results of Annexin V-FITC assay for apoptosis

**Figure 6:**
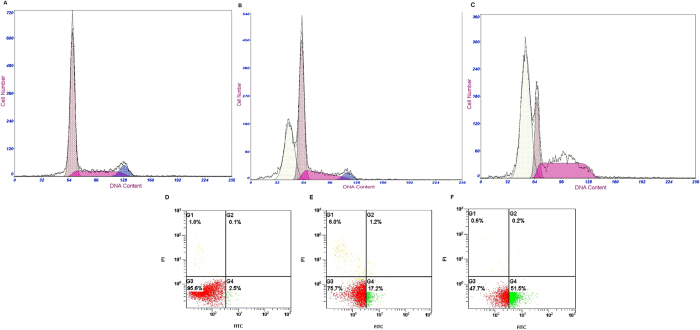
Annexin V-FITC flow cytometry. Cell cycle progression (**A**–**C**) and annexin V-FITC/PI staining was monitored in HepG2 cells following 24 h treatment with compound **Ik** at concentrations corresponding to their IC_50_ or 2 × IC_50_ (E and F). Representative dot plots of three independent experiments are given, presenting intact cells at lower-left quadrant, FITC(−)/PI(−); early apoptotic cells at lower-right quadrant, FITC(+)PI(−); late apoptotic or necrotic cells at upper-right quadrant, FITC(+)/PI(+); necrotic cells at upper-left quadrant, FITC(−)/PI(+).

**Figure 7:**
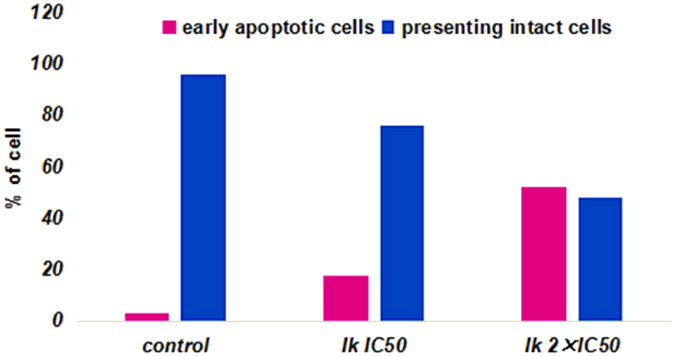
Apoptotic effect of compound. **Ik** was evaluated after 24 h treatment; bar graphs represent mean ± SD in at least three independent experiments.

Potential of the investigated compound **Ik** to induce apoptosis in HepG2 cells was analyzed by flow cytometry, following treatment with IC_50_ or 2 × IC_50_ concentrations for 24 h. The apoptotic effect of compound **Ik** was evaluated using Annexin V-FITC/PI dual staining assay, which can examine the occurrence of phosphatidylserine (PS) externalization as well as understand whether it is due to physiological apoptosis or nonspecific necrosis^22^. All data obtained in this study are presented in Figs 6 and 7, as a percentages of an early apoptotic cells, FITC(+)PI(−); late apoptotic cells, FITC(+)/PI(+); and necrotic cells, FITC(−)/PI(+); presenting intact cells, FITC(−)/PI(−).

Results revealed that compound **Ik** induced apoptotic changes following 24 h treatment. As shown in Figs 6 and 7, compound **Ik** initiated excellent apoptosis, in terms of FITC(+)PI(−) staining, compared to the control. Compound **Ik** exhibited 17.2% of apoptosis at IC_50_ and 51.5% of apoptosis at 2 × IC_50_ concentrations, whereas 2.5% of apoptosis was observed in control (0.1% DMSO). Besides, Compound **Ik** existed 75.7% of presenting intact cells at IC_50_ and 47.7% of presenting intact cells 2 × IC_50_ concentrations, while 95.6% of presenting intact cells was observed in control. From this experiment it was observed that the highly potential compound induced cell death in HepG2 cells at least partly (initially), by apoptosis. However, the precise mechanisms of cell death induction by tested compound still remain to be further explored.

### Structure and activity relationship (SAR)

Within the limits of experimental error, for the present series of compounds **Ia-f** and **IIa-f**, of which the N-protected group is carbobenzoxy, the compounds **Ia**, **Ib**, **Id**, **IIa**, **IIb** and **IId** displayed better antiproliferation activities. In terms of these compounds, the compounds with lower molecular weight indicated better inhibitory activities against the cell lines. On the contrary, when the N-protected group is tert-butoxycarbonyl, the other compounds **Il**, **Im**, **IIk** and **IIl** showed better inhibitory activities. It can be speculated, when steric hindrance is smaller, compounds bearing aromatic ring displayed better antiproliferation activities, otherwhise, compounds with lower molecular weight displayed better antitumor activities. As far as glycine and proline derivatives are concerned, N-carbobenzoxy protected target compounds displayed better inhibitory activities compared to N-*tert*-butoxycarbonyl-protected compounds. However, for phenylalanine and tryptophan derivatives, N-*tert*-butoxycarbonyl-protected compounds indicated better inhibitory activities. Meanwhile, we also can found that the compounds containing oxime unit presented poor antiproliferation activities than that of the compounds bearing hydrazine moiety, which perhaps to prove the importance of amide bond.

## Conclusion

In the present study, twenty-five novel peptidomimetics derivatives containing natural steroid moiety have been conveniently synthesized, and their potential antitumor activities have also been evaluated *in vitro*. The preliminary bioassay results indicated that some of the compounds displayed obviously good inhibition activities against human cancer cell lines including HepG2, A549 and A875, especially, compounds **Ii** and **Ik** exhibited high cytotoxic activities against the three cancer cell lines, which might be developed as novel lead scaffold for potential antitumor agents. The further annexin V-FITC assay revealed these highly potential compounds induced cell death in HepG2 cells at least partly by apoptosis.

## Experimental

### Synthesis of target compounds

The instrumentation, chemicals, synthetic procedures and characterization were provided in supplementary data.

### *In vitro* cytotoxicity assays

The *in vitro* cytotoxicity of the synthesized compounds against different human cancer cell lines (HepG2, A549, A875) was measured with the 3-(4,5-dimethylthiazol-2-yl)-2,5-diphenyl tetrazolium bromide (MTT) assay^21^. All the data of the experiment were analyzed with SPSS software, and the 50% inhibitory concentrations (IC_50_) of each compound for the different cell lines were determined. A control was run for each test, and all assays were performed in triplicate on three independent experiments, and measurement data were expressed as the mean ± S.D.

### Flow cytometric analysis of apoptosis

Quantitative analysis of apoptotic and necrotic cell death induced by the test compounds was performed by Annexin V-FITC apoptosis detection kit according to the manufacturer’s instructions (BD Biosciences). Briefly, 2 × 10^5^ HepG-2 cells were seeded in 6-well plates and grown overnight. After removal of the growth medium, cells were treated with compound **Ik** for 24 h, at concentrations corresponding to their IC_50s_ or 2 × IC_50s_. Cells treated with 0.1% DMSO were served as solvent control. Following treatment cells were harvested, washed twice with ice-cold PBS and resuspended in binding buffer. Then the cells were stained by adding 5 μL of Annexin V-FITC and 5 μL of propidium iodide, sit for 15 min at room temperature in the dark and analyzed by flow cytometry (Beckman coulter FC500).

## Additional Information

**How to cite this article**: Wang, X. *et al*. Peptidomimetics Based On Dehydroepiandrosterone Scaffold: Synthesis, Antiproliferation Activity, Structure-Activity Relationship, and Mechanisms. *Sci. Rep*. **6**, 32654; doi: 10.1038/srep32654 (2016).

## Supplementary Material

Supplementary Information

## Figures and Tables

**Figure 1:**
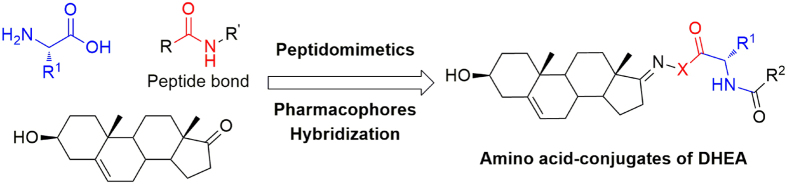
Design strategy of novel amino acid-conjugates of dehydroepiandrosterone.

**Table 1:**
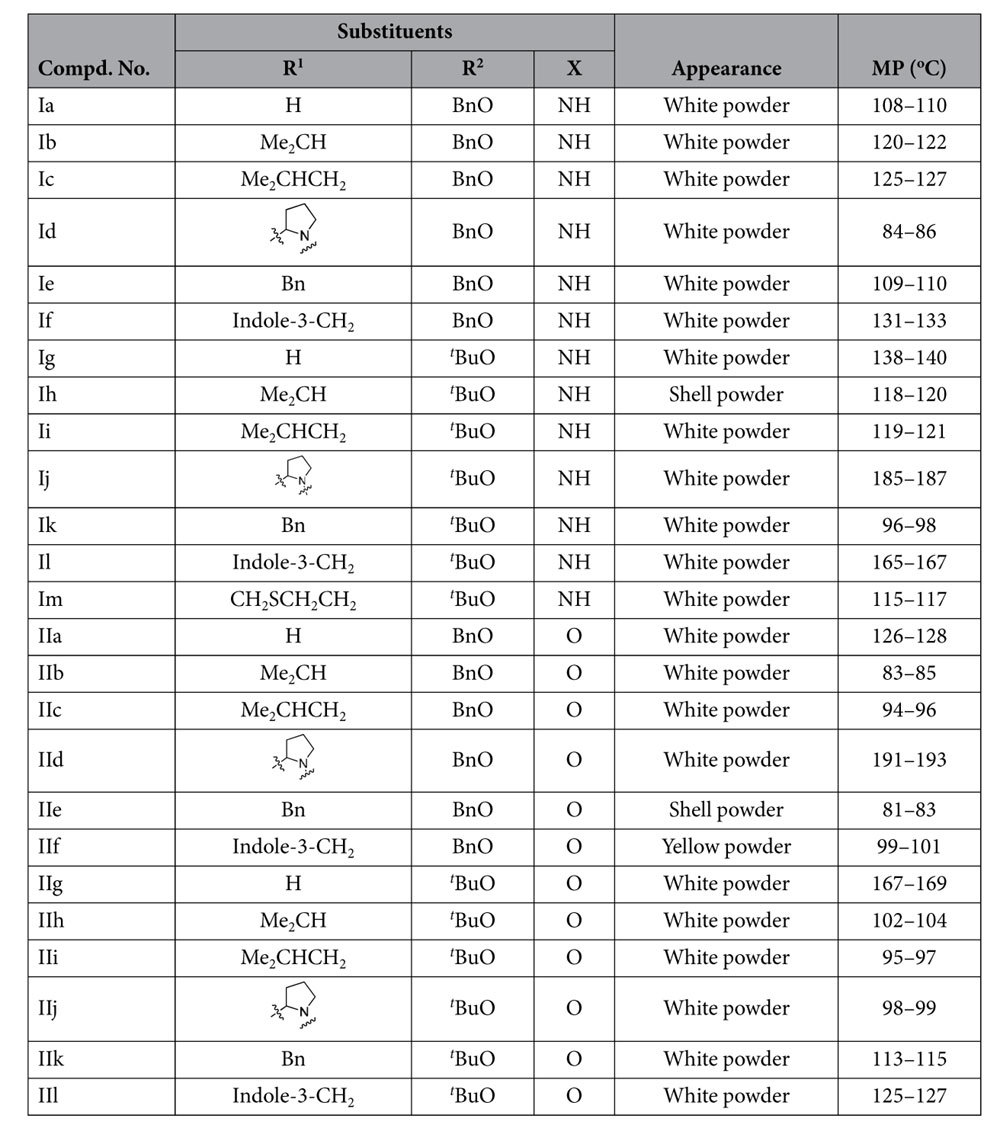
Chemical structure of synthesized peptidomimetics derivatives.

**Table 2:** Cytotoxic activity of the compounds against different human liver cells.

Entry	Compd. No.	*In vitro* cytotoxicity IC_50_^a^ (μM)		
		HepG2^b^	A549^b^	A875^b^
1	Ia	20 ± 4^c^	20 ± 3	18 ± 2
2	Ib	15 ± 4	17 ± 2	16 ± 6
3	Ic	14 ± 4	14 ± 3	14 ± 5
4	Id	12 ± 5	16 ± 3	19 ± 4
5	Ie	19 ± 4	22 ± 4	30 ± 8
6	If	>60	>60	>60
7	Ig	35 ± 5	32 ± 7	37 ± 1
8	Ih	26 ± 7	24 ± 6	27 ± 3
9	Ii	9 ± 3	10 ± 3	14 ± 3
10	Ij	24 ± 5	21 ± 2	24 ± 2
11	Ik	9 ± 2	6 ± 1	13 ± 1
12	Il	11 ± 4	8 ± 3	15 ± 6
13	Im	10 ± 2	10 ± 4	16 ± 5
14	IIa	21 ± 1	27 ± 6	29 ± 4
15	IIb	24 ± 7	19 ± 6	24 ± 0
16	IIc	29 ± 4	21 ± 5	17 ± 2
17	IId	7 ± 3	13 ± 4	15 ± 4
18	IIe	>60	37 ± 5	40 ± 2
19	IIf	28 ± 1	21 ± 4	29 ± 9
20	IIg	31 ± 6	23 ± 4	20 ± 2
21	IIh	23 ± 4	18 ± 2	14 ± 1
22	IIi	23 ± 7	23 ± 2	22 ± 5
23	IIj	22 ± 2	20 ± 2	18 ± 1
24	IIk	16 ± 2	16 ± 3	18 ± 2
25	IIl	17 ± 3	15 ± 2	15 ± 2
26	5-FU^*d*^	84 ± 25	115 ± 10	100 ± 24

^a^IC_50_ – Compound concentration required to inhibit tumor cell proliferation by 50%.

^b^Abbreviations: HepG2 – Human hepatocellular liver carcinoma cell line; A549 – Human lung cell line; A875 – human melanoma cell line.

^c^All assays were performed in triplicate on three independent experiments, and measurement data were expressed as the mean ± S.D.
