# Supplementary material for: Peptidomimetics Based On Dehydroepiandrosterone Scaffold: Synthesis, Antiproliferation Activity, Structure-Activity Relationship, and Mechanisms
Source: Sci Rep. 2016 Sep 2;6:32654. doi: 10.1038/srep32654 (PMC5009342; doi:10.1038/srep32654)
Supplement: Supplementary Information [file srep32654-s1.doc]

**Peptidomimetics Based On Dehydroepiandrosterone Scaffold: Synthesis, Antiproliferation Activity, Structure-Activity Relationship, and Mechanisms**

**Xiaohui Wang, Haihuan Su, Wenda Wang, Changshui Chen, Xiufang Cao***

*College of Science, Huazhong Agricultural University, Wuhan, 430070, China*

**Corresponding author: caoxiufang@mail.hzau.edu.cn*

**Supporting Information**

**Experimental Section**

1. **Instrumentation and chemicals**

All melting points (m.p.) were obtained with a digital model X-5 apparatus and are uncorrected. ^1^H NMR spectra were recorded on a Brucker spectrometer at 400 MHz with CDCl_3_ as the solvent and TMS as the internal standard; ^13^C NMR spectra were recorded on a Brucker spectrometer at 150 MHz with CDCl_3_ or DMSO-*d*_6_ as the solvent. Chemical shifts are reported in *δ* (parts per million) values. Coupling constants ^n^*J* are reported in Hz. Mass spectra were performed on a MicroMass Quattro micro^TMB^ API instrument. HRMS were recorded with Agilent-6224-TOF ESI/MS (ESI mode) instruments. Analytical thin-layer chromatography (TLC) was carried out on precoated plates, and spots were visualized with ultraviolet light. All chemicals or reagents used for syntheses were commercially available, were of AR grade, and were used as received. Anhydrous CH_2_Cl_2_ and CH_3_CN were dried according to standard methods. All other solvents and reagents were analytical reagent and used directly without purification.

1. **General synthetic procedure for** **2a-m**

A solution of di-*tert*-butyl pyrocarbonate (22 mmol) or benzyl carbonochloridate (22 mmol) in CH_2_Cl_2_ (4 mL) and NaOH (22 mmol) in H_2_O (4 mL) was added dropwise to an ice-cooled solution of amino acid (**1a-g**, 20 mmol) in aqueous solution of sodium hydroxide (22 mmol). Then the reaction was stirred at room temperature for several hours and monitored to the completion by thin-layer chromatography. The solution was washed with CH_2_Cl_2_, acidized, washed with ethyl acetate, sodium chloride aqueous solution and dried with anhydrous Na_2_SO_4_. The filtrate was evaporated under a reduced pressure to get N-substituted amino acids, which was used directly for the next reaction without further purification.

1. **General synthetic procedure for compound** **4**

A solution of dehydroepiandrosterone (20 mmol) in ethyl alcohol (20 ml) was added dropwise to hydrazine hydrate (22 mmol) at room temperature, then reflux at 78 ^o^C for about 6 hours. The solution was evaporated under a reduced pressure, dissolved in chloroform, washed with water and dried with anhydrous Na_2_SO_4_. The filtrate was evaporated under a reduced pressure to get compound 4, which was used directly for the next reaction without further purification.

1. **General synthetic procedure for compound** **5**

Dehydroepiandrosterone (20 mmol), [hydroxylamine](../../../../C:/Users/wxh/AppData/Local/Yodao/DeskDict/frame/20160112104120/javascript:void(0)%3B) [hydrochloride](../../../../C:/Users/wxh/AppData/Local/Yodao/DeskDict/frame/20160112104120/javascript:void(0)%3B) (22 mmol), [ethyl](../../../../C:/Users/wxh/AppData/Local/Yodao/DeskDict/frame/20160112104120/javascript:void(0)%3B) alcohol (20 ml) and water (20 ml) was added to bottle with two necks, stirred until balance. Then sodium acetate was added dropwise to the solution (20 mmol) at room temperature, and then the mixtures were heated to reflux and monitored by TLC. On completion of the reaction, the solution was filtrated under a reduced pressure. The residue was washed with water repeatedly to get compound **5**, which was used directly for the next reaction without further purification.

1. **General synthetic procedure for Ia-m and IIa-l**

The N-protected amino acids (1 mmol), N,N'-carbonyldiimidazole (1.2 mmol) and triethylamine (2 mmol) were located into an oven-dried round-bottomed flask equipped with a magnetic stir bar, and acetonitrile (20 mL) was then added. After 2 hour, the corresponding compounds 4 or 5 (1.1 mmol) was sequentially added and the reaction mixture was stirred at room temperature, and the reaction was detected by TLC. After completion of the reaction, the mixture was washed by acid, water and dried with anhydrous Na_2_SO_4_. The filtrate was evaporated under a reduced pressure and the residue was purified by silica gel column-chromatography (ethyl acetate/petroleum ether) or recrystallization to give target compounds **Ia-m** and **IIa-l**. Their physico-chemical properties and the spectra data are as follows:

5.1. Benzyl (2-((E)-2-((3S,10R,13S)-3-hydroxy-10,13-dimethyl-3,4,7,8,9,11,12,13,15,16 -decahydro-1H-cyclopenta[a]phenanthren-17(2H,10H,14H)-ylidene)hydrazinyl)-2-oxoethyl)carbamate **Ia**

This compound was obtained following the above method as white solid, mp: 108-110 ^o^C; ^1^H NMR (400 MHz, CDCl_3_): δ = 9.39 (s, 1H), 7.38-7.30 (m, 6H), 5.38 (d, *J* = 8Hz, 1H), 3.69 (s, 2H), 3.57-3.50 (s, 1H), 3.27 (s, 2H), 2.42-2.14 (m, 5H), 1.99-1.86 (m, 3H), 1.68-1.61 (m, 5H), 1.59-1.52 (m, 3H), 1.50-1.38 (m, 2H), 1.22-1.12 (m, 2H), 1.05 (s, 3H), 0.94 (s, 3H); ^13^C NMR (150 MHz, CDCl_3_): δ = 170.10, 166.57, 141.18, 137.65, 128.79, 128.70, 127.69, 120.82, 71.56, 59.23, 57.09, 53.59, 50.19, 44.54, 42.15, 37.15, 36.59, 33.64, 31.55, 31.29, 25.93, 23.50, 19.37, 16.56; ESI-MS: calcd for C_29_H_39_N_3_O_4_ ([M+Na^+^]^+^), 515.3; found, 515.2.

5.2 Benzyl (1-((E)-2-((3S,10R,13S)-3-hydroxy-10,13-dimethyl-3,4,7,8,9,11,12,13,15,16- decahydro-1H-cyclopenta[a]phenanthren-17(2H,10H,14H)-ylidene)hydrazinyl)-3-methyl-1-oxobutan-2-yl)carbamate **Ib**

This compound was obtained following the above method as white solid, mp: 120-122 ^o^C; ^1^H NMR (400 MHz, CDCl_3_): δ = 8.18 (s, 1H, N-H), 7.37-7.29 (m, 5H, Ar-H), 5.6 (d, *J* = 6Hz, 1H, N-H), 5.37 (d, *J* = 4Hz, 1H), 5.13-5.06 (m, 2H), 3.56-3.50 (m, 1H), 2.38-2.25 (m, 2H), 2.23-1.99 (m, 4H), 1.93-1.85 (m, 3H), 1.69-1.57 (m, 5H), 1.52-1.45 (m, 3H), 1.42-1.25 (m, 2H), 1.18-1.07 (m, 3H), 1.05-0.97 (m, 6H), 0.90 (s, 3H), 0.86 (s, 3H); ^13^C NMR (150 MHz, CDCl_3_): δ = 175.18, 168.23, 156.47, 141.05, 136.45, 128.47, 128.02, 127.80, 120.92, 71.55, 66.81, 58.90, 56.30, 53.65, 50.34, 44.58, 42.03, 37.14, 36.61, 33.73, 31.41, 31.26, 25.81, 23.53, 20.51, 19.42, 19.03, 17.35, 16.56; ESI-MS: calcd for C_32_H_45_N_3_O_4_ ([M+H]^+^), 536.3; found, 536.7; ESI-HRMS: calcd for C_32_H_45_N_3_O_4_ ([M+H]^+^), 536.34828; found, 536.34737.

5.3 Benzyl (1-((E)-2-((3S,10R,13S)-3-hydroxy-10,13-dimethyl-3,4,7,8,9,11,12,13,15,16- decahydro-1H-cyclopenta[a]phenanthren-17(2H,10H,14H)-ylidene)hydrazinyl)-4-methyl-1-oxopentan-2-yl)carbamate **Ic**

This compound was obtained following the above method as white solid, mp: 125-127 ^o^C. ^1^H NMR (400 MHz, CDCl_3_): δ = 7.99 (s, 1H, N-H), 7.36-7.28 (m, 5H, Ar-H), 5.45 (d, *J* = 8Hz, 1H), 5.37 (d, *J* = 4Hz, 1H), 5.21-5.11 (m, 2H), 3.57-3.51 (m, 1H), 2.38-2.25 (m, 3H), 2.22-1.99 (m, 3H), 1.94-1.85 (m, 3H), 1.77-1.66 (m, 3H), 1.63-1.50 (m, 6H), 1.47-1.36 (m, 3H), 1.34-1.10 (m, 3H), 1.07-1.01 (m, 6H), 0.95-0.90 (m, 6H); ^13^C NMR (150 MHz, CDCl_3_): δ = 174.70, 167.23, 156.21, 140.96, 136.35, 128.46, 127.96, 120.78, 71.54, 66.70, 53.66, 50.40, 50.31, 44.59, 42.14, 37.18, 36.62, 34.06, 31.51, 31.26, 24.81, 23.63, 23.51, 21.52, 20.57, 19.42, 16.69; ESI-MS: calcd for C_33_H_47_N_3_O_4_ ([M+H]^+^), 550.4; found, 550.7; ESI-HRMS: calcd for C_33_H_47_N_3_O_4_ ([M+H]^+^), 550.36393; found, 550.36273.

5.4 Benzyl 2-((E)-2-((3S,10R,13S)-3-hydroxy-10,13-dimethyl-3,4,7,8,9,11,12,13,15,16- decahydro-1H-cyclopenta[a]phenanthren-17(2H,10H,14H)-ylidene)hydrazinecarbonyl)pyrrolidine-1-carboxylate **Id**

This compound was obtained following the above method as white solid, mp: 84-86 ^o^C; ^1^H NMR (400 MHz, CDCl_3_): δ = 9.73 (s, 1H, N-H), 7.35-7.26 (m, 5H, Ar-H), 5.35 (d, *J* = 4Hz, 1H), 5.20-5.06 (m, 2H), 4.39 (d, *J* = 8Hz, 1H), 3.67-3.57 (m, 1H), 3.52-3.38 (m, 2H), 2.36-2.21 (m, 4H), 2.15-2.05 (m, 2H), 1.98-1.91 (m, 3H), 1.88-1.76 (m, 4H), 1.66-1.57 (m, 5H), 1.52-1.35 (m, 3H), 1.16-1.08 (m, 3H), 1.05 (s, 3H), 0.89 (s, 3H); ^13^C NMR (150 MHz, CDCl_3_): δ = 171.07, 166.35, 156.37, 141.16, 136.29, 128.50, 128.21, 127.60, 120.82, 71.52, 67.49, 59.01, 57.93, 53.63, 50.18, 47.01, 44.26, 42.15, 37.16, 36.54, 33.66, 31.24, 23.41, 20.49, 19.41, 16.52; ESI-MS: calcd for C_32_H_43_N_3_O_4_ ([M+H]^+^), 534.3; found, 534.4; ESI-HRMS: calcd for C_32_H_43_N_3_O_4_ ([M+H]^+^), 534.33263; found, 534.33167.

5.5 Benzyl (1-((E)-2-((3S,10R,13S)-3-hydroxy-10,13-dimethy l-3,4,7,8,9,11,12,13, 15,1 6 -decahydro-1H-cyclopenta[a]phenanthren-17(2H,10H,14H)-ylidene)hydrazinyl)-1-oxo-3-phenylpr

opan-2-yl)carbamate **Ie**

This compound was obtained following the above method as white solid, mp: 109-110 ^o^C;^1^H NMR (400 MHz, CDCl_3_): δ = 8.50 (s, 1H, N-H), 7.35-7.30 (m, 5H, Ar-H), 7.25-7.15 (m, 5H, Ar-H), 5.37 (d, *J* = 8Hz, 1H), 5.39 (d, *J* = 4Hz, 2H), 5.10 (d, *J* = 4Hz, 1H), 5.07 (d, *J* = 4Hz, 1H), 5.37-5.32 (m, 1H), 3.23-3.11 (m, 1H), 2.99-2.94 (m, 1H), 2.35-2.26 (m, 3H), 1.91-1.85 (m, 4H), 1.73-1.61 (m, 5H), 1.59-1.35 (m, 5H), 1.18-1.09 (m, 3H), 1.07-1.87 (m, 6H); ^13^C NMR (150 MHz, CDCl_3_): δ = 172.71, 167.88, 155.61, 141.11, 136.48, 129.55, 128.45, 128.24, 127.96, 126.67, 120.89, 71.53, 66.45, 53.63, 53.00, 50.36, 44.50, 42.12, 38.09, 37.26, 36.60, 33.96, 31.26, 25.64, 23.51, 20.52, 19.43, 16.60; ESI-MS: calcd for C_36_H_45_N_3_O_4_ ([M+H]^+^), 584.3; found, 584.5.

5.6 Benzyl (1-((E)-2-((3S,10R,13S)-3-hydroxy-10,13-dimethyl-3,4,7,8,9,11,12,13,15,16 -decahydro-1H-cyclopenta[a]phenanthren-17(2H,10H,14H)-ylidene)hydrazinyl)-3-(1H-indol-3-yl)

-1-oxopropan-2-yl)carbamate **If**

This compound was obtained following the above method as white solid, mp: 131-133 ^o^C;^1^H NMR (400 MHz, CDCl_3_): δ = 8.15 (s, 1H, N-H), 8.02 (s, 1H, N-H), 7.60 (d, *J* = 8Hz, 1H, Ar-H), 7.35-7.30 (m, 6H, Ar-H), 7.21-7.02 (m, 3H, Ar-H), 5.66 (d, *J* = 8Hz, 1H, N-H), 5.47-5.42 (q, *J* = 8Hz ,1H), 5.39-5.32 (m, 1H), 5.14-5.08 (m, 2H), 3.58-3.51 (m, 1H), 3.37-3.21 (m, 2H), 2.36-2.24 (m, 3H), 2.02-1.95 (m, 2H), 1.91-1.84 (m, 4H), 1.68-1.59 (m, 5H), 1.54-1.41 (m, 4H), 1.19-1.08 (m, 2H), 1.06-0.80 (m, 6H); ^13^C NMR (150 MHz, CDCl_3_): δ = 173.63, 167.92, 155.81, 141.08, 136.51, 136.14, 128.53, 128.45, 128.02, 121.59, 120.93, 119.25, 118.83, 111.23, 71.56, 66.67, 53.49, 50.27, 44.50, 42.12, 37.15, 36.60, 33.72, 31.50, 31.22, 23.45, 20.52, 19.38, 16.53,16.38; ESI-MS: calcd for C_38_H_46_N_4_O_4_ ([M+H]^+^), 624.4; found, 624.6.

5.7 tert-Butyl (2-((E)-2-((3S,10R,13S)-3-hydroxy-10,13-dimethyl-3,4,7,8,9,11,12,13,15,16- ecahydro-1H-cyclopenta[a]phenanthren-17(2H,10H,14H)-ylidene)hydrazinyl)-2-oxoethyl)carbamate **Ig**

This compound was obtained following the above method as white solid, mp: 138-140 ^o^C;^1^H NMR (400 MHz, CDCl_3_): δ = 8.05 (s, 1H), 5.37 (d, *J* = 4Hz, 1 H), 4.28 (t, *J* = 4Hz, 8Hz, 1H), 3.58-3.50 (m, 1H), 2.37-2.28 (m, 2H), 2.25-2.01 (m, 3H), 1.97-1.87 (m, 4H), 1.64-1.53 (m, 8H), 1.47 (s, 9H), 1.36-1.11 (m, 5H), 1.05 (s, 3H), 0.88 (s, 3H); ^13^C NMR (150 MHz, CDCl_3_): δ = 171.23, 167.34, 155.98, 141.10, 120.88, 79.58, 71.54, 53.63, 50.28, 44.48, 42.13, 37.16, 36.61, 33.86, 31.50, 31.26, 28.35, 25.52, 23.50, 20.49, 19.42, 16.72; ESI-MS: calcd for C_26_H_41_ N_3_O_4_ ([M+H]^+^), 460.3; found, 460.5; ESI-HR MS: calcd for C_26_H_41_ N_3_O_4_ ([M+H]^+^), 460.31698; found, 460.31582.

5.8 tert-Butyl (1-((E)-2-((3S,10R,13S)-3-hydroxy-10,13-dimethyl-3,4,7,8,9,11,12,13,15,16- decahydro-1H-cyclopenta[a]phenanthren-17(2H,10H,14H)-ylidene)hydrazinyl)-3-methyl-1-oxobu

tan-2-yl)carbamate **Ih**

This compound was obtained following the above method as shell solid, mp: 118-120 ^o^C;^1^H NMR (400 MHz, CDCl_3_): δ = 8.14 (s, 1H, N-H), 5.37 (d, *J* = 8Hz, 1H), 5.31 (d, *J* = 4Hz, 1H, N-H) , 5.03 (dd, *J* = 4Hz, 1H), 3.58-3.51 (m, 1H), 2.43-2.25 (m, 3H), 2.18-1.99 (m, 4H), 1.95-1.86 (m, 3H), 1.70-1.63 (m, 3H), 1.58-1.49 (m, 3H), 1.45 (s, 9H), 1.37-1.29 (m, 2H), 1.0-1.10 (m, 3H), 1.04-0.85 (m, 12H); ^13^C NMR (150 MHz, CDCl_3_): δ = 173.98, 166.65, 155.97, 141.08, 120.93, 79.07, 71.63, 55.76, 53.75, 50.44, 44.51, 42.20, 37.20, 36.67, 33.79, 31.59, 31.32, 30.82, 29.70, 28.38, 25.37, 23.54, 20.54, 19.72, 19.43, 16.58; ESI-MS:calcd for C_29_H_47_N_3_O_4_ ([M+H]^+^), 502.4; found, 502.5.

5.9 tert-Butyl (1-((E)-2-((3S,10R,13S)-3-hydroxy-10,13-dimethyl-3,4,7,8,9,11,12,13,15, 16-decahydro-1H-cyclopenta[a]phenanthren-17(2H,10H,14H)-ylidene)hydrazinyl)-4-methyl-1-oxopentan-2-yl)carbamate **Ii**

This compound was obtained following the above method as white solid, mp: 119-121 ^o^C; ^1^H NMR (400 MHz, CDCl_3_): δ = 8.26 (s, 1H, N-H), 5.37 (d, *J* = 4Hz, 1H ), 5.17-5.09 (q, 1H, N-H), 4.93-4.87 (m, 1H), 3.57-3.51 (m, 1H), 2.48 -2.20 (m, 3H), 2.19-2.02 (m, 2H), 1.95-1.85 (m, 4H), 1.78-1.62 (m, 4H), 1.60-1.47 (m, 6H), 1.45 (s, 9H), 1.38-1.10 (m, 4H), 1.05-1.0 (m, 6H), 0.97-0.90 (m, 6H); ^13^C NMR (150 MHz, CDCl_3_): δ = 175.47, 167.16, 155.50, 140.77, 120.58, 79.31, 71.10, 53.67, 50.31, 49.83, 44.59, 42.00, 37.14, 36.62, 34.07, 31.27, 28.36, 24.58, 23.66, 22.89, 21.54, 20.58, 19.42, 16.66; ESI-MS: calcd for C_30_H_49_N_3_O_4_ ([M+H]^+^), 516.4; found, 516.5.

5.10 tert-Butyl 2-((E)-2-((3S,10R,13S)-3-hydroxy-10,13-dimethyl-3,4,7,8,9,11,12,13,15,16- decahydro-1H-cyclopenta[a]phenanthren-17(2H,10H,14H)-ylidene)hydrazinecarbonyl)pyrrolidine-1-carboxylate **Ij**

This compound was obtained following the above method as white solid, mp: 185-187 ^o^C;^1^H NMR (400 MHz, CDCl_3_): δ = 10.04 (s, 1H, N-H), 5.37 (d, *J* = 4Hz, 1H), 4.36 (d, *J* = 8Hz, 1H), 3.57-3.52 (m, 1H), 3.49-3.34 (m, 2H), 2.62 (s, 1H), 2.50-2.39 (m, 1H), 2.34-2.12 (m, 5H), 2.09-2.01 (m, 2H), 1.96 -1.86 (m, 7H), 1.76-1.53 (m, 5H), 1.48 (s, 9H), 1.34-1.23 (m, 1H), 1.21-1.10 (m, 2H), 1.04 (s, 3H), 0.94-0.87 (t, *J* = 8Hz, 3H); ^13^C NMR (150 MHz, CDCl_3_): δ = 170.94, 167.69, 156.51, 140.78, 120.93, 80.79, 79.46, 71.48, 58.56, 57.30, 53.66, 51.70, 50.23, 44.21, 42.10, 37.15, 36.60, 33.62, 31.44, 31.30, 28.43, 26.59, 23.40, 20.50, 19.41, 16.57; ESI-MS: calcd for C_29_H_45_N_3_O_4_ ([M+H]^+^), 500.3; found, 500.6; ESI-HRMS: calcd for C_29_H_45_N_3_O_4_ ([M+H]^+^), 500.34828; found, 500.34790.

5.11 tert-Butyl (1-((E)-2-((3S,10R,13S)-3-hydroxy-10,13-dimethyl-3,4,7,8,9,11,12,13,15,16 -decahydro-1H-cyclopenta[a]phenanthren-17(2H,10H,14H)-ylidene)hydrazinyl)-1-oxo-3-phenylpropan-2-yl)carbamate **Ik**

This compound was obtained following the above method as white solid, mp: 96-98 ^o^C; ^1^H NMR (400 MHz, CDCl_3_): δ = 8.21 (s, 1H, N-H), 7.31-7.27 (m, 2H, Ar-H), 7.22-7.16 (m, 3H, Ar-H), 5.37 (d, *J* = 4Hz, 1H), 5.34-5.29 (m, 1H), 5.23 (d, *J* = 8Hz, 1H, N-H), 3.57-3.51 (m, 1H), 3.21-3.17 (m, 1H), 3.14-3.04 (m, 1H), 2.38-2.24 (m, 3H), 2.19-2.03 (m, 4H), 1.97-1.88 (m, 3H), 1.85-1.77 (m, 3H), 1.71-1.62 (m, 4H), 1.59-1.50 (m, 3H), 1.41 (d, *J* = 16Hz, 9H), 1.08-1.04 (m, 3H), 0.92-0.85 (m, 3H); ^13^C NMR (150 MHz, CDCl_3_) δ = 173.47, 167.75, 155.27, 140.90, 135.13, 129.54, 128.90, 127.46, 120.94, 71.57, 57.19, 53.54, 51.71, 50.16, 47.56, 44.12, 42.14, 37.14, 36.61, 35.85, 31.45 , 30.75, 21.87, 20.33, 19.42, 16.43, 13.54; ESI-MS: calcd for C_33_H_47_N_3_O_4_ ([M+H]^+^), 550.4; found, 550.4.

5.12 tert-Butyl (1-((E)-2-((3S,10R,13S)-3-hydroxy-10,13-dimethyl-3,4,7,8,9,11,12,13,15,16 -decahydro-1H-cyclopenta[a]phenanthren-17(2H,10H,14H)-ylidene)hydrazinyl)-3-(1H-indol-3-yl)-1-oxopropan-2-yl)carbamate **Il**

This compound was obtained following the above method as white solid, mp: 165-167 ^o^C;^1^H NMR (400 MHz,CDCl_3_): δ = 8.36 (s, 1H), 8.19 (d, *J* = 12Hz, 1H), 7.65 (q, 1H), 7.35 (dd, *J* = 8Hz, 1H), 7.21-7.05 (m, 3H), 5.39-5.33 (m, 3H), 3.58-3.51 (m, 1H), 3.37-3.16 (m, 2H), 2.36-2.23 (m, 4H), 2.01-1.94 (m, 2H), 1.90-1.83 (m, 5H), 1.71-1.61 (m, 4H), 1.56-1.48 (m, 5H), 1.43 (s, 9H), 1.05-0.79 (m, 6H)); ^13^C NMR (150 MHz, DMSO-*d*_6_): δ = 174.33, 167.34, 155.68, 141.86, 136.44, 127.80, 121.28, 120.63, 119.14, 118.35, 111.73, 110.57, 78.22, 70.44, 53.22, 52.17, 50.30, 44.35, 42.66, 37.34, 36.67, 34.26, 31.86, 31.35, 28.63, 23.43, 20.71, 19.64, 17.03; ESI-MS: calcd for C_35_H_48_N_4_O_4_ ([M+H]^+^), 589.4; found, 589.5; ESI-HRMS: calcd for C_35_H_48_N_4_O_4_ ([M+H]^+^), 589.37483; found, 589.37412.

5.13 tert-Butyl (1-((E)-2-((3S,10R,13S)-3-hydroxy-10,13-dimethyl-3,4,7,8,9,11,12,13,15,16- decahydro-1H-cyclopenta[a]phenanthren-17(2H,10H,14H)-ylidene)hydrazinyl)-4-(methylthio)-1-oxobutan-2-yl)carbamate **Im**

This compound was obtained following the above method as white solid, mp: 115-117 ^o^C;^1^H NMR (400 MHz, CDCl_3_): δ = 8.16 (s, 1H, N-H), 5.44 (d, *J* = 16Hz, 1H, N-H), 5.37 (d, *J* = 4Hz, 1H), 5.11 (s, 1H), 3.57-3.51 (m, 1H), 2.64-2.56 (m, 2 H), 2.40-2.20 (m, 4H), 2.20-2.15 (m, 2H), 2.10 (s, 3H), 2.03-1.95 (m, 3H), 1.91-1.83 (m, 4H), 1.71-1.58 (m, 4H), 1.54-1.51 (m, 3H), 1.46 (s, 9H), 1.20-1.11 (m, 2H), 1.05 (s, 3H), 0.91 (s, 3H); ^13^C NMR (150 MHz, CDCl_3_): δ = 173.30, 167.29, 155.55, 141.10, 120.85, 79.62, 71.51, 53.67, 51.14, 50.29, 44.56, 42.10, 37.15, 36.62, 33.88, 32.73, 31.47, 31.23, , 28.34, 23.53, 20.50, 19.42, 16.61, 15.57, 15.17; ESI-MS: calcd for C_29_H_47_N_3_O_4_S ([M+H]^+^), 534.3; found, 534.5; ESI-HRMS: calcd for C_29_H_47_N_3_O_4_S ([M+H]^+^), 534.33600; found, 534.33514.

5.14 Benzyl (2-(((E)-((3S,10R,13S)-3-hydroxy-10,13-dimethyl-3,4,7,8,9,11,12,13,15,16- decahydro-1H-cyclopenta[a]phenanthren-17(2H,10H,14H)-ylidene)amino)oxy)-2-oxoethyl)carbamate **IIa**

This compound was obtained following the above method as white solid, mp: 126-128 ^o^C; ^1^H NMR (400 MHz, CDCl_3_): δ = 7.42 (d, *J* = 4Hz, 2H, Ar-H), 7.33 (t, *J* = 4Hz, 8Hz, 3H, Ar-H), 7.25 (d, *J* = 4Hz, 1H, N-H), 5.38 (d, *J* = 4Hz, 1H), 3.89 (s, 3H), 3.58-3.51 (m, 1 H), 3.41 (s, 1H), 2.64-2.57 (m, 1H), 2.52-2.43 (m, 1H), 2.35-2.22 (m, 2H), 2.15-2.04 (m, 2H), 1.90-1.80 (m, 3H), 1.72-1.60 (m, 4H), 1.56-1.36 (m, 5H), 1.22-1.12 (m, 2 H),1.05 (s, 3H), 1.02 (s, 3H). ESI-MS: calcd for C_29_H_38_N_2_O_5_ ([M +Na+H]^+^), 518.3; found, 518.8.

5.15 Benzyl (1-(((E)-((3S,10R,13S)-3-hydroxy-10,13-dimethyl-3,4,7,8,9,11,12,13,15,16- decahydro-1H-cyclopenta[a]phenanthren-17(2H,10H,14H)-ylidene)amino)oxy)-3-methyl-1-oxobutan-2-yl)carbamate **IIb**

This compound was obtained following the above method as white solid, mp: 83-85 ^o^C;^1^H NMR (400 MHz, CDCl_3_): δ = 7.37-7.30 (m, 5H), 5.37 (d, *J* = 4Hz, 2H), 5.12 (s, 2H), 4.40-4.36 (m, 1H), 3.57-3.48 (m, 1H), 2.67-2.52 (m, 1H), 2.35-2.20 (m, 2H), 2.18-2.00 (m, 3H), 1.95-1.85 (m, 4H), 1.69-1.59 (m, 4H), 1.55-1.50 (m, 3H), 1.44-1.24 (m, 2H), 1.27-1.14 (m, 2H), 1.04-0.96 (m, 12H); ^13^C NMR (150 MHz, DMSO-*d*_6_): δ = 170.32, 168.30, 157.04, 141.84, 136.73, 128.81, 128.20, 120.64, 70.43, 65.83, 60.16, 54.14, 53.61, 50.35, 47.66, 43.33, 42.69, 37.33, 36.68, 34.55, 31.88, 31.29, 30.10, 25.31, 23.33, 22.12, 20.65, 19.48, 18.45, 17.45; ESI-MS: calcd for C_32_H_44_N_2_O_5_ ([M+H]^+^), 537.3; found, 537.2; ESI-HRMS: calcd for C_32_H_44_N_2_O_5_ ([M+Na]^+^), 559.31424; found, 559.31203.

5.16 Benzyl (1-((E)-2-((3S,10R,13S)-3-hydroxy-10,13-dimethyl-3,4,7,8,9,11,12,13,15,16- decahydro-1H-cyclopenta[a]phenanthren-17(2H,10H,14H)-ylidene)hydrazinyl)-4-methyl-1-oxopentan-2-yl)carbamate **IIc**

This compound was obtained following the above method as white solid, mp: 94-96 ^o^C; ^1^H NMR (400 MHz, CDCl_3_): δ = 7.35-7.39 (m, 5H), 5.36 (d, *J* = 8Hz, 1H), 5.21 (d, *J* = 8Hz, 1H, N-H), 5.10 (s, 2H), 4.51-4.50 (m, 1H), 3.56-3.50 (m, 1H), 2.63-2.49 (m, 2H), 2.34-2.21 (m, 2H), 2.17-1.93 (m, 3H), 1.88-1.74 (m, 4H), 1.69-1.56 (m, 6H), 1.54-1.44 (m, 4H), 1.42-1.33 (m, 2H), 1.27-1.13 (m, 2H), 1.03-0.94 (m, 12H); ^13^C NMR (150 MHz, DMSO-*d*_6_): δ = 179.59, 168.30, 156.35, 141.82, 137.40, 128.81, 128.25, 128.15, 120.63, 70.42, 65.80, 54.13, 50.34, 44.98, 43.32, 42.67, 37.32, 36.66, 34.53, 31.86, 31.28, 25.30, 24.76, 23.31, 20.64, 19.62, 17.43; ESI-MS: calcd for C_30_H_48_N_2_O_5_ ([M+Na]^+^), 573.4; found, 573.6; ESI-HRMS: calcd for C_30_H_48_N_2_O_5_ ([M+Na]^+^), 573.32989; found, 573.32945.

5.17 Benzyl 2-((((E)-((3S,10R,13S)-3-hydroxy-10,13-dimethyl-3,4,7,8,9,11,12,13,15,16- decahydro-1H-cyclopenta[a]phenanthren-17(2H,10H,14H)-ylidene)amino)oxy)carbonyl)pyrrolidine-1-carboxylate **IId**

This compound was obtained following the above method as white solid, mp: 191-193 ^o^C; ^1^H NMR (400 MHz, CDCl_3_): δ = 7.36-7.39 (m, 5H), 5.35 (d, *J* = 8 Hz, 1H), 5.21-5.09 (m, 3H), 4.44 (s, 1H), 3.62-3.45 (m, 3H), 2.62-2.45 (m, 1H), 2.30-2.09 (m, 7H), 2.01-1.83 (m, 7H), 1.67-1.60 (m, 4H), 1.54-1.40 (m, 4H), 1.02-0.92 (m, 6H); ^13^C NMR (150 MHz, CDCl_3_): δ = 175.90, 166.39, 156.80, 140.87, 128.41, 127.88, 121.12, 71.66, 54.13, 50.27, 49.17, 43.79, 42.24, 37.20, 36.62, 33.95, 31.60, 31.28, 25.62, 24.94, 23.23, 20.58, 19.42, 16.94; ESI-MS: calcd for C_32_H_42_N_2_O_5_ ([M+Na]^+^), 557.3; found, 556.8; ESI-MS: calcd for C_32_H_42_N_2_O_5_ ([M+Na]^+^), 557.29859; found, 557.29875.

5.18 Benzyl (1-(((E)-((3S,10R,13S)-3-hydroxy-10,13-dimethyl-3,4,7,8,9,11,12,13,15,16- decahydro-1H-cyclopenta[a]phenanthren-17(2H,10H,14H)-ylidene)amino)oxy)-1-oxo-3-phenylpropan-2-yl)carbamate **IIe**

This compound was obtained following the above method as shell solid, mp: 81-83 ^o^C; ^1^H NMR (400 MHz, CDCl_3_): δ = 7.35-7.14 (m, 10H), 5.36 (d, *J* = 8Hz,1H), 5.21 (d, *J* = 8 Hz, 1H), 5.13-5.08 (m, 2H), 4.68 (s, 1H), 3.56-3.50 (m, 1H), 3.24-3.09 (m, 2H), 2.61-2.46 (m, 3H), 2.34-2.21 (m, 3H), 2.11-2.04 (m, 1H), 1.94-1.85 (m, 4H), 1.70-1.57 (m, 4H), 1.50-1.35 (m, 3H), 1.25-1.10 (m, 2H), 1.04-0.94 (m, 6H); ^13^C NMR (150 MHz, DMSO-*d*_6_): δ = 173.68, 168.37, 156.46, 141.84, 138.39, 137.37, 129.56, 128.77, 128.63, 128.19, 127.97, 126.83, 120.65, 70.44, 65.70, 56.04, 54.15, 50.35, 43.33, 42.65, 37.32, 36.93, 36.67, 34.53, 31.84, 31.28, 25.31, 23.32, 20.65, 19.63, 17.42; ESI-MS: calcd for C_36_H_44_N_2_O_5_ ([M+Na]^+^), 607.3; found, 607.4.

5.19 Benzyl (1-(((E)-((3S,10R,13S)-3-hydroxy-10,13-dimethyl-3,4,7,8,9,11,12,13,15,16- decahydro-1H-cyclopenta[a]phenanthren-17(2H,10H,14H)-ylidene)amino)oxy)-3-(1H-indol-3-yl)-1-oxopropan-2-yl)carbamate **IIf**

This compound was obtained following the above method as yellow solid, mp: 99-101 ^o^C; ^1^H NMR (400 MHz, CDCl_3_): δ = 8.04 (s, 1H, N-H), 7.54 (d, *J* = 8Hz, 1H, Ar-H), 7.30 (s, 5H, Ar-H), 7.46-7.33 (m, 4H, Ar-H), 5.35-5.29 (m, 2H), 5.13-5.04 (q, *J* = 12Hz, 2H), 4.73 (d, *J* = 8Hz, 1H), 3.55-3.45 (m, 1H), 3.41-3.27 (m, 2H), 2.61-2.20 (m, 4H), 1.91-1.83 (m, 4H), 1.65-1.49 (m, 4H), 1.46-1.35 (m, 5H), 1.18-1.05 (m, 3H), 1.03 (s, 3H), 0.92 (s, 3H); ^13^C NMR (150 MHz, DMSO-*d*_6_): δ = 174.26, 168.37, 156.48, 141.20, 137.42, 136.56, 128.79, 128.05, 124.22, 121.39, 120.65, 118.86, 111.89, 110.54, 70.45, 65.76, 55.42, 54.15, 50.35, 43.34, 42.67, 37.33, 36.68, 34.55, 31.87, 31.28, 27.36, 25.32, 23.32, 20.65, 19.63, 17.43, 15.98; ESI-MS: calcd for C_38_H_45_N_3_O_5_ ([M+Na]^+^), 646.3; found, 646.5.

5.20 tert-Butyl (2-(((E)-((3S,10R,13S)-3-hydroxy-10,13-dimethyl-3,4,7,8,9,11,12,13,15,16- decahydro-1H-cyclopenta[a]phenanthren-17(2H,10H,14H)-ylidene)amino)oxy)-2-oxoethyl)carbamate **IIg**

This compound was obtained following the above method as white solid, mp: 167-169 ^o^C; ^1^H NMR (400 MHz, CDCl_3_): δ = 5.37 (d, *J* = 4Hz, 1H), 5.12 (s, 1H, N-H), 4.07 (d, *J* = 8Hz, 2H), 3.56-3.51 (m, 1H), 2.70-2.52 (m, 1H), 2.32-2.22 (m, 2H), 2.10-2.05 (m, 2H), 1.88-1.81 (m, 3H), 1.68-1.48 (m, 6H), 1.46 (s, 9H), 1.42-1.06 (m, 6H), 1.02 (d, *J* = 8Hz, 6H); ^13^C NMR (150 MHz, CDCl_3_): δ = 179.17, 169.41, 155.71, 141.02, 120.81, 80.06, 71.51, 53.96, 49.94, 45.15, 42.10, 41.75, 37.13, 36.54, 33.46, 31.47, 31.26, 28.30, 27.22, 23.10, 20.41, 19.41, 16.76; ESI-MS: calcd for C_26_H_40_N_2_O_5_ ([M+Na]^+^), 483.3; found, 483.5.

5.21 tert-Butyl (1-(((E)-((3S,10R,13S)-3-hydroxy-10,13-dimethyl-3,4,7,8,9,11,12,13,15,16- decahydro-1H-cyclopenta[a]phenanthren-17(2H,10H,14H)-ylidene)amino)oxy)-3-methyl-1-oxobutan-2-yl)carbamate **IIh**

This compound was obtained following the above method as white solid, mp: 102-104 ^o^C; ^1^H NMR (400 MHz, CDCl_3_): δ = 5.36 (d, *J* = 4Hz, 1H), 5.11 (d, *J* = 8Hz, 1H), 4.31-4.26 (m, 1H), 3.56-3.49 (m, 1H), 2.62-2.50 (m, 1H), 2.33-2.14 (m, 2H), 2.11-1.95 (m, 2H), 1.91-1.84 (m, 3H), 1.68-1.58 (m, 3H), 1.54-1.45 (m, 5H), 1.41 (s, 9H), 1.29-1.21 (m, 3H), 1.14-1.05 (m, 2H), 1.03-0.94 (m, 12H); ^13^C NMR (150MHz, DMSO-*d*_6_): δ = 174.03, 168.26, 156.25, 141.83, 120.62, 78.41, 70.43, 59.57, 54.15, 50.36, 43.32, 42.69, 37.33, 36.67, 34.55, 31.88, 31.29, 29.97, 28.67, 25.30, 23.33, 20.66, 19.63, 19.01, 18.61, 17.44, 17.07; ESI-MS: calcd for C_29_H_46_N_2_O_5_ ([M+Na]^+^), 525.3; found, 525.5.

5.22 tert-Butyl (1-(((E)-((3S,10R,13S)-3-hydroxy-10,13-dimethyl-3,4,7,8,9,11,12,13,15,16- decahydro-1H-cyclopenta[a]phenanthren-17(2H,10H,14H)-ylidene)amino)oxy)-4-methyl-1-oxopentan-2-yl)carbamate **IIi**

This compound was obtained following the above method as white solid, mp: 95-97 ^o^C; ^1^H NMR (400 MHz, CDCl_3_): δ = 5.36 (d, *J* = 4Hz, 1H), 4.98-4.89 (m, 1H), 4.37 (d, 1H), 3.57-3.49 (m, 1H), 2.66-2.47 (m, 3H), 2.34-2.21 (m, 3H), 2.13-2.04 (m, 3H), 1.88-1.83 (m, 3H), 1.77-1.58 (m, 6H), 1.56-1.49 (m, 3H), 1.44 (d, *J* = 8Hz, 9H), 1.25-1.13 (m, 2H), 1.02 (d, *J* = 8Hz, 6H), 0.95 (d, *J* = 4Hz, 6H); ^13^C NMR (150 MHz, DMSO-*d*_6_): δ = 175.18, 168.29, 156.06, 141.82, 120.63, 78.36, 70.42, 54.13, 52.21, 50.34, 43.32, 42.67, 37.32, 36.67, 34.54, 31.87, 31.28, 28.66, 25.30, 24.79, 23.36, 21.65, 20.64, 19.63, 17.44; ESI-MS: calcd for C_30_H_48_N_2_O_5_ ([M+Na]^+^), 539.4; found, 539.5.

5.23 tert-Butyl 2-((((E)-((3S,10R,13S)-3-hydroxy-10,13-dimethyl-3,4,7,8,9,11,12,13,15,16- decahydro-1H-cyclopenta[a]phenanthren-17(2H,10H,14H)-ylidene)amino)oxy)carbonyl)pyrrolidine-1-carboxylate **IIj**

This compound was obtained following the above method as white solid, mp: 98-99 ^o^C; ^1^H NMR (400 MHz, CDCl_3_): δ = 5.36 (d, *J* = 4Hz, 1H), 4.34 (s,1H), 4.25 (s, 1H), 3.56-3.34 (m, 4H), 2.61-2.42 (m, 2H), 2.34-2.20 (m, 4H), 2.11-2.03 (m, 2H), 1.96-1.84 (m, 7H), 1.66-1.52 (m, 5H), 1.48-1.41 (m, 9H), 1.21-1.09 (m, 2H), 1.03 (s, 3H), 0.92 (s, 3H); ^13^C NMR (150 MHz, DMSO-*d*_6_): δ = 174.86, 168.28, 153.56, 141.84, 120.64, 79.05, 70.44, 59.12, 54.16, 50.37, 46.55, 43.33, 42.69, 37.34, 36.68, 34.56, 31.88, 31.30, 31.27, 30.74, 29.87, 28.58, 28.40, 25.30, 24.35, 23.61, 20.66, 19.63, 17.44; ESI-MS: calcd for C_29_H_44_N_2_O_5_ ([M+Na]^+^), 523.3; found, 523.6.

5.24 tert-Butyl (1-(((E)-((3S,10R,13S)-3-hydroxy-10,13-dimethyl-3,4,7,8,9,11,12,13,15,16- decahydro-1H-cyclopenta[a]phenanthren-17(2H,10H,14H)-ylidene)amino)oxy)-1-oxo-3-phenylpropan-2-yl)carbamate **IIk**

This compound was obtained following the above method as white solid, mp: 113-115 ^o^C; ^1^H NMR (400 MHz, CDCl_3_): δ = 7.31-7.28 (m, 2 H), 7.24-7.17 (m, 3H), 5.36 (s, 1H), 5.08 (d, *J* = 12Hz, 1H), 4.66 (d, *J* = 8Hz, 1H), 3.57-3.49 (m, 1H), 3.08 (d, *J* = 8Hz, 2H), 2.60-2.39 (m, 2H), 2.36 -2.21 (m, 4H), 1.95-1.76 (m, 4H), 1.69-1.55 (m, 4H), 1.53-1.45 (m, 3H), 1.41 (s, 9H), 1.21-1.06 (m, 3H), 1.03-0.94 (m, 6H); ^13^C NMR (150 MHz, DMSO-*d*_6_): δ = 174.06, 168.28, 155.90, 141.86, 138.52, 129.55, 128.58, 126.75, 120.61, 78.48, 70.45, 55.61, 54.17, 50.39, 43.33, 42.70, 37.35, 36.69, 34.57, 31.89, 31.32, 28.62, 25.29, 23.33, 20.67, 19.63, 17.44; ESI-MS: calcd for C_33_H_46_N_2_O_5_ ([M+Na]^+^), 573.3; found, 573.3.

5.25 tert-Butyl (1-(((E)-((3S,10R,13S)-3-hydroxy-10,13-dimethyl-3,4,7,8,9,11,12,13,15,16- decahydro-1H-cyclopenta[a]phenanthren-17(2H,10H,14H)-ylidene)amino)oxy)-3-(1H-indol-3-yl)-1-oxopropan-2-yl)carbamate **IIl**

This compound was obtained following the above method as white solid, mp: 125-127 ^o^C; ^1^H NMR (400 MHz, CDCl_3_): δ = 8.08 (s, 1H, N-H), 7.55 (d, *J* = 8Hz, 1H, Ar-H), 7.32-7.26 (m, 1H, Ar-H), 7.17-7.03 (m, 3H, Ar-H), 5.33 (d, *J* = 4Hz, 1H), 5.12 (d, *J* = 8Hz, 1H, N-H), 4.76-4.71 (m, 1H), 3.54-3.48 (m, 1H), 3.28 (d, *J* = 4Hz, 2H), 2.32-2.16 (m, 3H), 2.08-2.00 (m, 2H), 1.85 (d, *J* = 12Hz, 2H), 1.70-1.55 (m, 7H), 1.51-1.46 (m, 3H), 1.43 (s, 9H), 1.26-1.09 (m, 3H), 1.03-0.95 (m, 6H); ^13^C NMR (150 MHz, DMSO-*d*_6_): δ = 179.37, 170.63, 155.86, 141.81, 136.57, 127.49, 124.42, 121.41, 120.50, 118.90, 111.84, 110.05, 78.78, 70.41, 60.24, 54.39, 53.60, 49.93, 44.89, 42.66, 37.31, 36.62, 33.78, 31.88, 31.23, 28.62, 26.82, 23.06, 21.25, 20.57, 19.61, 17.02, 14.56; ESI-MS: calcd for C_35_H_47_N_3_O_5_ ([M+Na]^+^), 612.4; found, 612.5; ESI-HR MS: calcd for C_35_H_47_N_3_O_5_ ([M+K]^+^), 628.31473; found, 628.31610.
